# Texture perception at the foot sole: comparison between walking, sitting, and to the hand

**DOI:** 10.1152/jn.00170.2024

**Published:** 2024-07-17

**Authors:** Luke D. Cleland, Mia Rupani, Celia R. Blaise, Toby J. Ellmers, Hannes P. Saal

**Affiliations:** ^1^Active Touch Laboratory, Department of Psychology, University of Sheffield, Sheffield, United Kingdom; ^2^Insigneo Institute for In Silico Medicine, University of Sheffield, Sheffield, United Kingdom; ^3^Neuroscience Institute, University of Sheffield, Sheffield, United Kingdom; ^4^Cognitive Studies, Department of Philosophy, University of Sheffield, Sheffield, United Kingdom; ^5^Centre for Vestibular Neurology, Department of Brain Sciences, Imperial College London, London, United Kingdom

**Keywords:** foot, psychophysics, texture, touch

## Abstract

We frequently interact with textured surfaces with both our feet and hands. Like texture’s importance for grasping, texture perception via the foot sole might provide important signals about the stability of a surface, aiding in maintaining balance. However, how textures are perceived by the foot, and especially under the high forces experienced during walking, is unknown. The current study builds on extensive research investigating texture perception at the hand by presenting everyday textures to the foot while stepping onto them, exploring them with the foot while sitting, and exploring them with the hand. Participants rated each texture along three perceptual dimensions: roughness, hardness, and stickiness. Participants also rated how stable their posture felt when standing upon each texture. Results show that perceptual ratings of each textural dimension were highly correlated across conditions. Hardness exhibited the greatest consistency and stickiness the weakest. Moreover, correlations between stepping and exploration with the foot were lower than those between exploration with the foot and exploration with the hand, suggesting that mode of interaction (high vs. low force) impacts perception more than body region used (foot vs. hand). On an individual level, correlations between conditions were higher than those between participants, suggesting that differences are greater between individuals than between mode of interaction or body region. When investigating the relationship to perceived stability, only hardness contributed significantly, with harder surfaces rated as more stable. Overall, tactile perception appears consistent across body regions and interaction modes, although differences in perception are greater during walking.

**NEW & NOTEWORTHY** We frequently interact with textured surfaces using our feet, but little is known about how textures on the foot sole are perceived as compared with the hand. Here, we show that roughness, hardness, and stickiness ratings are broadly consistent when stepping on textures, exploring them with the foot sole, or with the hand. Hardness also contributes to perceived stability.

## Graphical abstract

**Figure:**
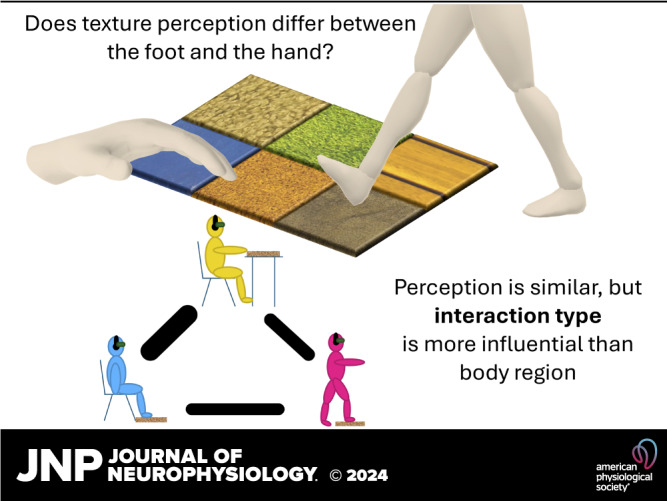

## INTRODUCTION

Imagine waking up in the middle of the night and needing to use the bathroom. Navigating through the darkness and relying on our sense of touch, our feet effectively detect subtle differences in texture, allowing us to differentiate between the rough fabric of the bedroom carpet, the hardwood floor in the hallway, and the smooth surface of the bathroom tile. Are such judgments made through our feet comparable with texture percepts arising from our hands? The feet differ from the hands in their tactile innervation, their mechanical behavior, and in how they typically interact with surfaces, but how perceptually relevant these factors are is hard to answer. The vast majority of research on texture perception has focused on the hands and comparatively little is known about whether and how texture perception differs across body parts, though some differences between hairless and hairy skin have been established (1).

Both the palmar hand surface and the foot sole are hairless skin and therefore show similar innervation characteristics. Notably, though, the foot sole contains only a quarter of the number of tactile afferents compared with the hand (2), yielding markedly lower spatial acuity (3). This might affect the perception of roughness, which relies on a spatial code for coarser textures (4). Indeed, the perception of roughness has been found to differ across hand regions (5), suggesting afferent density contributes to differences in texture perception.

There are also differences in the mechanics of the skin between the hand and the foot. Specifically, skin on the foot sole is thicker (6), harder, and more variable, than palmar skin (7, 8). It has been shown that skin stiffness directly affects discrimination accuracy for the softness of different materials (9), with greater stiffness leading to poorer compliance discrimination. This suggests that perception at the foot may be poorer compared with the hand due to differences in skin mechanics.

Finally, one of the largest differences between natural interactions with the external world involving our hands and feet is the mode of interaction. Texture exploration using the hands involves relatively low forces (10) and typically includes lateral movement between the skin and the surface (11, 12), whereas the most common mode of interaction with the foot is arguably during walking, which involves much higher forces and less lateral movement. The force applied to a surface is especially important when judging roughness (13, 14), with higher forces leading to greater roughness ratings (14, 15). As the foot regularly experiences forces exceeding three times body mass (16, 17), materials may be perceived as much rougher at the foot sole than at the hand.

Investigating texture perception on the foot sole is important, because textures might provide important clues about the stability of a given surface. For example, in the hand, textures have direct functional consequences for the effective interactions with objects, enabling us to hold objects without them slipping by applying the appropriate force to ensure optimal friction between our fingers and the object (18, 19). The ability to distinguish between surfaces is equally important at our feet, to be able to inform us of the surface we are standing or walking on. Decoding such information allows humans to walk on stable surfaces and adjust gait to prevent falls, for example if the ground is soft or slippery (20). Research has also suggested that increased tactile feedback from the foot sole aids balance: for example, standing on surfaces that contain small textured elements reduces participants’ natural sway (21–23), even though these surfaces are not inherently more stable. This effect is exploited by textured insoles that aim to improve balance (24, 25). It has been suggested that presenting textures to the foot sole will increase tactile feedback, resulting in greater information relating to shifts in pressure (26). In turn, these cues will improve balance and reduce the risk of falling (27). However, which textures might be especially suited for such purpose is not entirely clear. In the hand, surface roughness is highly correlated with neural activity (28), suggesting that roughness might be a good proxy to identify suitable textures.

Here, we investigate how everyday textures on the foot sole are perceived compared with the hand. Participants rated textures along three perceptual dimensions: roughness, hardness, and stickiness, which are among the most prominent in texture studies focusing on the hand (29, 30). Our aim was to study texture perception during natural behaviors in which textures are typically encountered. Therefore, when testing the hand participants gently explored the texture with their hand while seated. In contrast, when testing the foot participants stepped onto and off each texture patch to mimic texture perception during standing and walking. To test whether any putative differences in perception were due to the body part or due to the different mode of interaction, we included a third condition where people explored the texture gently with their foot while sitting, mirroring texture exploration on the hand. Thus, if exploration with the hand and foot yield similar ratings, but differ from those when stepping onto the texture, then perceptual judgments are driven by how textures are interacted with and not the identity of the body parts. Conversely, to the extent that both conditions involving the foot yield similar ratings, but differ from the hand, then differences between those body parts (innervation, skin mechanics) and not different modes of interaction are the main drivers of perception.

## MATERIALS AND METHODS

### Participants

Twenty young, healthy participants (7 males, 13 females) with a mean age of 20.00 (2.66) yr with no history of sensory deficits were recruited to take part in the study. One participant did not complete the exploration condition with the hand, and therefore the within-participant comparisons between the hand and foot conditions are not analyzed for this participant. All participants provided written informed consent prior to the start of data collection. The study protocol was approved by the ethical review board of the Department of Psychology at the University of Sheffield (Protocol No. 052209).

### Textures

**Figure 1.**
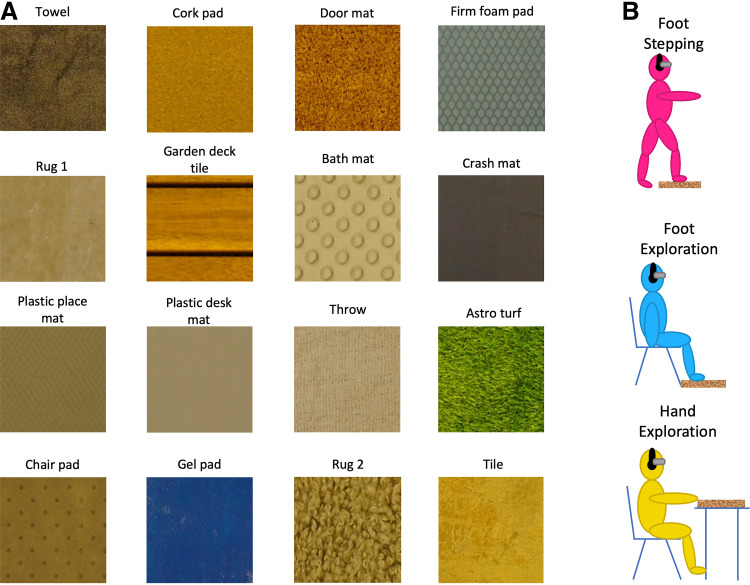
Overview of the textures and presentation conditions. *A*: a section (roughly 10 × 10 cm) of each of the 16 everyday textures used, in the experimental grid layout. *B*: illustration of the three presentation conditions, stepping with the foot (*top*), exploration with the foot (*middle*), and exploration with the hand (*bottom*).

Sixteen everyday textures were included in the experiment (Fig. 1; see Table 1 for details). The textures selected are commonly experienced by the foot sole and were expected to vary across perceptual dimensions. They included those experienced in the household such as rugs, those experienced outside such as artificial grass and garden decking, along with materials commonly used during insole manufacturing such as cork and gel. All texture patches were at least 25 by 22 cm in size to allow participants to step onto and stand on a given patch with both feet. The same texture samples were used across all conditions.

**Table 1.** Texture properties

Texture Number	Texture Name	Material
1	Door mat	99.5% coir, 5% polyester
2	Rug 1	100% polyester
3	Towel	100% cotton
4	Rug 2	100% polyester
5	Cork pad	Cork, polyurethane unbleached paper
6	Plastic place mat	Polypropylene plastic, polyethylene plastic, synthetic rubber
7	Chair pad	back: 100% polypropylene, inner: polyurethane foam 30 kg/m^3^
8	Bath mat	Natural rubber, calcium carbonate
9	Astro turf	Nylon, polypropylene or polyethylene
10	Plastic desk mat	Polyethylene plastic, EVA plastic
11	Garden deck tile	Acacia
12	Tile	Ceramic
13	Crash mat	Outer: nylon (230 gsm soft nylon polyester PU coated water-resistant fabric), inner: polyurethane foam (5 cm reconstituted foam + 5 cm medium density foam)
14	Firm foam pad	100% foamed EVA
15	Gel pad	Polyurethane elastic fiber
16	Throw	40% lyocell, 39% acrylic, 21% polyester

### Experimental Protocol

Participants took part in three presentation conditions: “Foot-Stepping,” where participants stepped onto and off of the texture, “Foot-Exploration,” where participants explored the textures with the foot sole while sitting, and “Hand-Exploration,” where participants explored the textures with the hand while sitting. In all conditions, participants wore a blindfold and noise-canceling headphones throughout the experimental session to remove visual and auditory influences on perception. During the stepping condition, participants were guided at all times using a guiding stick, receiving a tap on the hand to ask participants to step onto and off of each of the textures, which were laid out on the floor in a 4 × 4 grid. Participants were instructed to lead with their dominant foot and make a cautious step onto the texture before pausing for 2–3 s on the texture with both feet and stepping off. As soon as participants stepped off of the texture, they stated their rating of the texture along the dimension in question (see next paragraph). For the exploration conditions with the foot or hand, participants remained seated and textures were presented by the research team in front of their foot or hand. Between each presentation, participants rested their feet on a foot-rest on the chair or their hands on the edge of the table. Participants received a tap on the leg or hand to instruct them to begin, and finish, exploring the texture, stating their rating following exploration termination. As in the stepping condition, in the exploration conditions, participants were free to use both feet or hands. This protocol ensured that each texture was presented for between 2 and 3 s for all three conditions.

Participants judged each texture along one of three textural dimensions—roughness, hardness, and stickiness—in separate blocks, using free magnitude scaling, a method commonly used in previous texture research (11, 13, 31–33). For example, for roughness they were instructed to “rate the subjective roughness using any positive number including zero, with low numbers indicating very smooth and high numbers indicating very rough surfaces.” Participants were also given an example prior to the start of the first presentation: “If you rate the first surface as a three, and the second surface is twice as rough then it would be a six.” The same instructions were provided for all three textural dimensions, with the wording adjusted for each dimension. For the stepping condition only, participants were asked in a separate experimental block to rate the perceived stability of each textured surface. Participants rated “how stable you feel when stepping onto, and off of, the texture. If you feel so unstable that you would fall, rate it as zero.” Self-rated perceived (in)stability has been shown to correlate highly with actual postural sway values in healthy controls (34, 35), particularly when eyes are closed (35), as in the present study. At the end of the block, participants were asked to rate how stable they would feel in “two-foot stance on a flat surface, feet shoulder width apart and holding onto a rail” for a comparison value against all textures. The experimenter demonstrated this stance to aid the participant in obtaining the stance.

Each experimental block contained three presentations of any given texture. A separate experimental block was run for each combination of interaction mode (3) and textural dimension (3), yielding 16 × 3 × 3 × 3 = 432 textural ratings and 16 × 3 = 48 stability ratings in total for each participant. To speed up the experimental protocol and to minimize potential familiarization with the textures, the three interaction modes were run in a fixed order: first stepping, which was presumed the least sensitive condition, followed by exploration with the foot and finally exploration by the hand. Textures were presented in a different pseudo-random order for each of the three textural dimension blocks, and for the stability block. The same order was kept between all blocks for a given perceptual dimension and across conditions to control for order effects and facilitate data collection via a fixed texture grid in the stepping condition. A full experiment typically took ∼90 min to complete.

### Statistical Analysis

All analysis was run in Python, using Pandas (v.2.0.3), Scipy (v.1.10.1), and Statsmodels (v.0.13.5). As no strict rating scale was provided, participants were free to provide scores with no upper limit constraining perceptual ratings. Using free magnitude scaling allows participants to use a scale that feels natural for them, without being limited or biased through the use of an example of a maximum score. To ensure ratings could be interpreted across participants, each rating was normalized by dividing by the mean rating for its originating experimental block. This normalization preserves the ratio of ratings between textures, while setting the mean rating to 1 for each participant. To investigate whether participants’ ratings changed between presentations in the first presentation condition (roughness while stepping) due to potential habituation or adjustment to the texture variety, repeated-measures ANOVAs were conducted for all 16 textures. There were no significant changes in rating over the course of this presentation block. Perceptual ratings were then averaged across the three repeats in each experimental block, generating a “mean ratio” for each texture-condition-dimension-participant combination, with this value used for all further analyses. Textures were then ranked based on this rating. This study aimed to identify differences between texture ranks, and therefore implemented nonparametric analyses to investigate such differences.

For the group-level analysis, normalized perceptual ratings were averaged across all participants and Spearman’s Rho correlations were then calculated between conditions.

For the participant-level analysis, within-participant Spearman’s Rho correlations were calculated for each participant. Repeated-measures *t* tests were conducted on these correlations to identify whether perceptual ratings were more similar across some conditions than others.

To investigate how similar perceptual ratings were across participants, Spearman’s Rho correlations were calculated between every pair of participants (190) for each condition and textural dimension (6). To investigate whether there were significant differences between correlations coefficients between-condition and between-participants, each *r* value was transformed using Fisher’s z-transformation before being compared using two-sample *t* tests.

Repeated measures, nonparametric Friedman’s tests were run to identify whether there were significant differences in texture ranks on a given perceptual dimension between conditions. A significant Friedman’s test was followed up by nonparametric post hoc Wilcoxon tests, with a Bonferroni correction applied to account for multiple comparisons, yielding a corrected *P* value of 0.016.

To test whether the fact that some textures showed differences in their perceptual ratings across multiple textural dimensions could be explained by chance, we ran a Monte Carlo simulation over 100,000 trials: we randomly sampled textures from three sets independently and calculated how often these textures cooccurred at the same or a greater rate than found experimentally to generate a *P* value for the null hypothesis assuming independence.

To investigate the relationship between the perception of textural properties and participants’ perception of stability, a linear regression was run to investigate the contribution of the three textural properties (roughness, hardness, and stickiness) in explaining perception of stability ratings during stepping onto and off of each texture.

## RESULTS

We presented 16 textures to 20 participants across three different conditions: “Foot-Stepping,” where participants stepped onto and off a texture with bare feet; “Foot-Exploration,” where participants explored the textures with their feet while sitting; and finally “Hand-Exploration,” where participants explored the textures with their hands (see materials and methods for details). In each condition, participants judged the roughness, hardness, or stickiness of the textures in separate experimental blocks using free magnitude scaling.

### Group-Level Results

As the different interaction conditions were run in separate blocks, most of the subsequent analysis will consider each texture’s rank for the textural dimension in question to allow meaningful comparisons across conditions. In a first analysis, we averaged textural ratings after normalizing them across all participants and then compared their ranks across interaction conditions.

**Figure 2.**
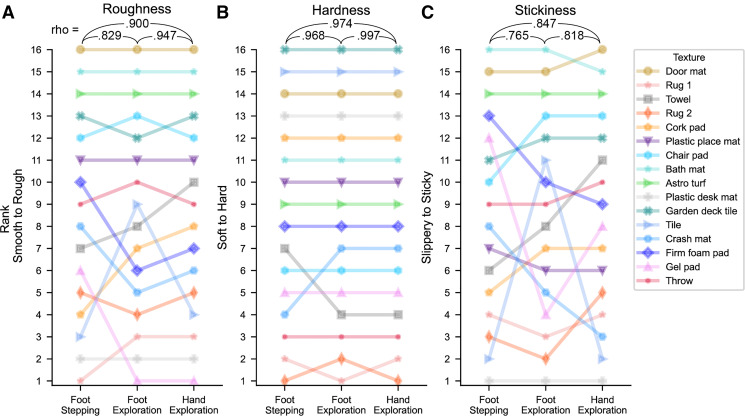
Group-level perceptual ratings across different interaction conditions. Slope charts showing texture ranks calculated from normalized perceptual ratings averaged across participants for roughness (*A*), hardness (*B*), and stickiness (*C*). Spearman’s Rho correlations between conditions displayed at the top of each plot. All correlations are significant with *P* < 0.001.

We found that perceptual ratings for each textural dimension were highly correlated across conditions (Fig. 2). Hardness was the most highly correlated dimension (all *r* > 0.96), with texture rankings nearly identical whether participants stepped onto them, or explored them with their feet or hands (Fig. 2*B*). For roughness, perception of very rough materials was consistent across conditions, although there was greater disagreement for smoother textures. Correlations were lower between the two foot conditions compared with the exploration conditions, though overall agreement was still high (all *r* > 0.82). Stickiness ratings were the least consistent across conditions (all *r* > 0.76), with one texture (tile) moving by nine ranks when explored with the foot.

Overall, although textures were generally ranked similarly, independent of body region or mode of contact, the correlation between the two foot conditions was sometimes lower than the correlation between the foot and hand exploration conditions, suggesting a stronger impact of mode of interaction on perception than body region.

### Consistency within and across Participants

Next, we investigated whether and how the responses of individual participants were correlated between conditions and with those of other participants.

**Figure 3.**
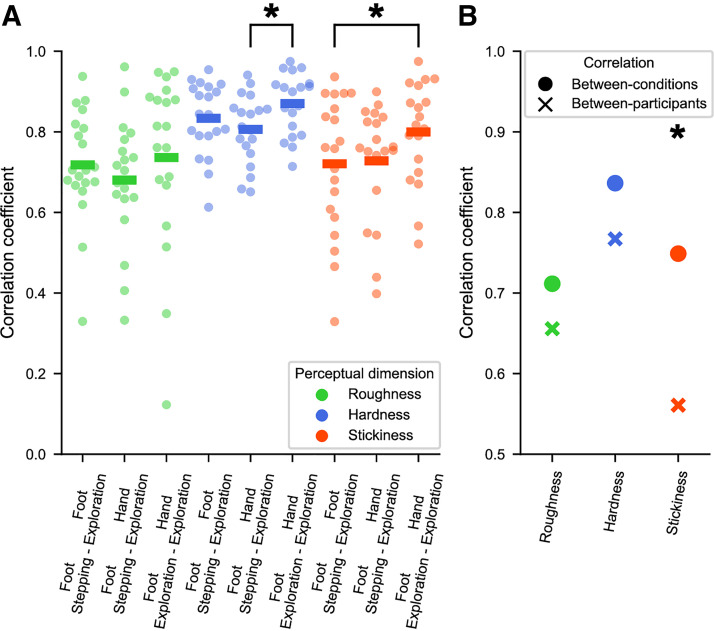
Between-participant and between-condition correlations. *A*: between-condition correlations for each participant. Each point represents the correlation in perceptual ratings between two conditions for a given participant. The horizontal line represents the mean correlation across all participants. *Significant repeated-measures *t* test (*P* < 0.050), indicating a difference in the between-condition correlation coefficients. *B*: comparison of average between-participant (crosses) and between-condition (circles) correlations for each perceptual dimension. *Significant difference.

The participant-level results mirrored the group-level ones in that perceptual ratings were generally highly correlated across different conditions (Fig. 3*A*). Again, the correlation between the hand and foot exploration was in some cases higher than the correlations involving the stepping condition (foot/hand exploration vs. stepping/hand exploration for hardness: *t* = 3.69, *P* = 0.002; foot/hand exploration vs. stepping/foot exploration for stickiness: *t* = 2.30, *P* = 0.034; see Fig. 3*A*). This suggests a stronger impact of mode of interaction on perception than body region, when there were differences between these conditions.

Notably, average participant-level correlations between conditions were greater than those between participants (Fig. 3*B*). Comparing the correlation coefficients across participants with those obtained within participants (see materials and methods), stickiness ratings were significantly less consistent between participants than between interaction conditions within the same participants (*t* = 2.39, *P* = 0.017). Although the same pattern exists for roughness (*t* = 0.74, *P* = 0.462) and hardness (*t* = 1.38, *P* = 0.169), the differences between were not statistically significant. Thus, texture perception is at least as consistent between body regions and modes of contact within a given participant, than it is between participants in the same condition.

### Differences between Interaction Conditions

**Figure 4.**
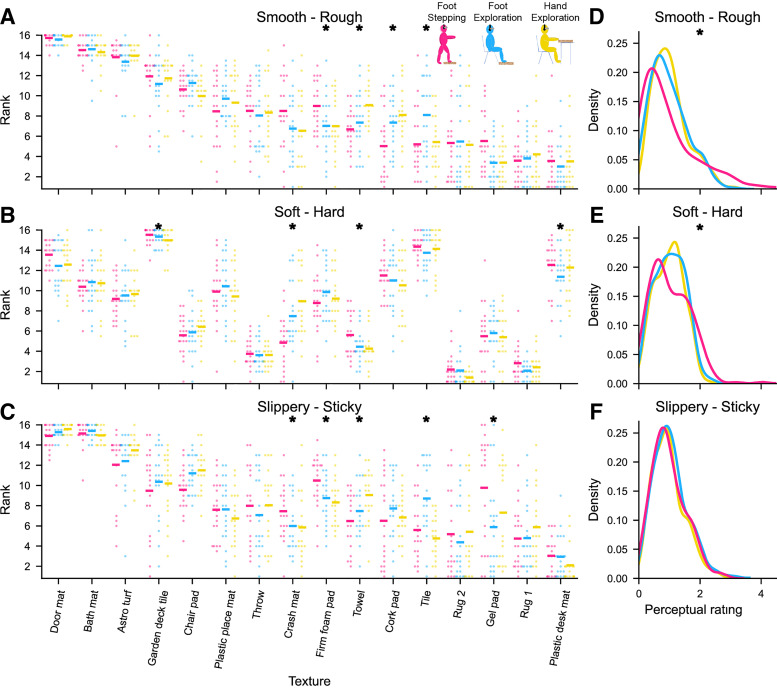
Differences in perception between conditions. *Left*: participant rankings for each texture for roughness (*A*), hardness (*B*), and stickiness (*C*), sorted by mean roughness rating. Mean ranks are indicated by horizontal lines. Individual points denote ranks for single participants. *Significant Friedman’s tests showing a difference in ranks between conditions. *Right*: kernel density plots showing the distribution of responses for roughness (*D*), hardness (*E*), and stickiness (*F*). *Significant Levene’s tests for equal variance.

Next, we further investigated differences between interaction conditions, by testing whether texture ranks differed significantly between conditions. For roughness, Friedman’s tests revealed significant differences for four textures in the middle of the smoothness-roughness spectrum (Fig. 4*A*). Post hoc tests revealed that almost all differences occurred between the stepping condition and either the foot or hand exploration conditions. Only one significant difference, for the tile, occurred between the foot and hand exploration conditions (see Table 2 for details). For hardness, Friedman’s tests revealed significant differences in rank for four textures (Fig. 4*B*), though post hoc tests were only significant for two textures. Again, most of the differences involved the stepping condition. Finally, for stickiness perception, significant differences were found for five textures (Fig. 4*C*), with these spread relatively equally across conditions. Overall, as differences more often involved the stepping condition, this might suggest that they were driven more by the mode of interaction (low vs. high force) rather than the fact that a different body region was involved (hand vs. foot), in agreement with our earlier observations.

**Table 2.** Post hoc Wilcoxon tests run on textures with significant Friedman’s test

Dimension	Texture	FS-FE	FS-HE	FE-HE
Roughness	Firm foam pad	33.5**	31.5*	85.5
	Towel	63.5	24.5**	49.5
	Cork pad	36.5*	23.5**	69.0
	Tile	18.0**	66.0	32.5**
Hardness	Garden deck tile	34.5	23.0	44.0
	Crash mat	3.0**	1.0**	34.5*
	Towel	23.0*	32.0*	48.0
	Plastic desk mat	46.0	48.0	47.0
Stickiness	Crash mat	34.0*	47.0	59.0
	Firm foam pad	38.0*	22.0**	77.5
	Towel	32.5	21.0**	35.0*
	Tile	22.0**	73.0	21.0**
	Gel pad	13.0**	37.0	28.0*

Statistics show *W* value. FS, foot-stepping; FE, foot-exploration; HE, hand-exploration. *P < 0.050; **significant following Bonferroni correction for multiple comparisons, *P* < 0.016. All significant values (marked as * or **) are rendered in bold.

Notably, when a texture was judged differently across conditions, this often occurred along multiple textural dimensions: the towel yielded significant differences across all three textural dimensions, and the tile, firm foam pad, and crash mat differed across two dimensions each. Indeed, Monte Carlo simulations confirmed that these cooccurrences were unlikely to arise when assuming independence across textural dimensions *P* = 0.006, see materials and methods for details), suggesting that a texture yielding a perceptual difference across one textural dimension was likely to do so across another.

So far, our analysis has focused on texture ranks, rather than their ratings directly. Since ratings were collected in different blocks for different interaction conditions, they might not be directly comparable. However, we can instead investigate whether the spread of responses, e.g., how much rougher the roughest texture feels compared with the smoothest one, differs across conditions. We found that the spread of perceptual scores differed between presentation conditions for roughness and hardness (Levene’s tests of equality of variance; *F* = 17.63, *P* < 0.001 and *F* = 15.36, *P* < 0.001, respectively, Fig. 4, *D* and *E*). Specifically, perceptual roughness ratings during stepping were spread further (Fig. 4*D*) compared with foot and hand exploration. On average, the roughest texture was rated as just over 10 times rougher than the smoothest texture during stepping. In contrast, during foot and hand exploration, the roughest texture rated at just over, and just under, five times as rough as the smoothest texture, respectively. This suggests that roughness levels were magnified when walking, making textures seem rougher than when perceiving them with the hand or under low forces during exploration with the foot. The same pattern was evident for hardness perception, with increased spread of the responses during stepping. In contrast, the distribution of responses for stickiness perception was equal across all conditions (*F* = 0.37, *P* = 0.692, Fig. 4*F*).

### Textural Dimensions and Stability Ratings

**Figure 5.**
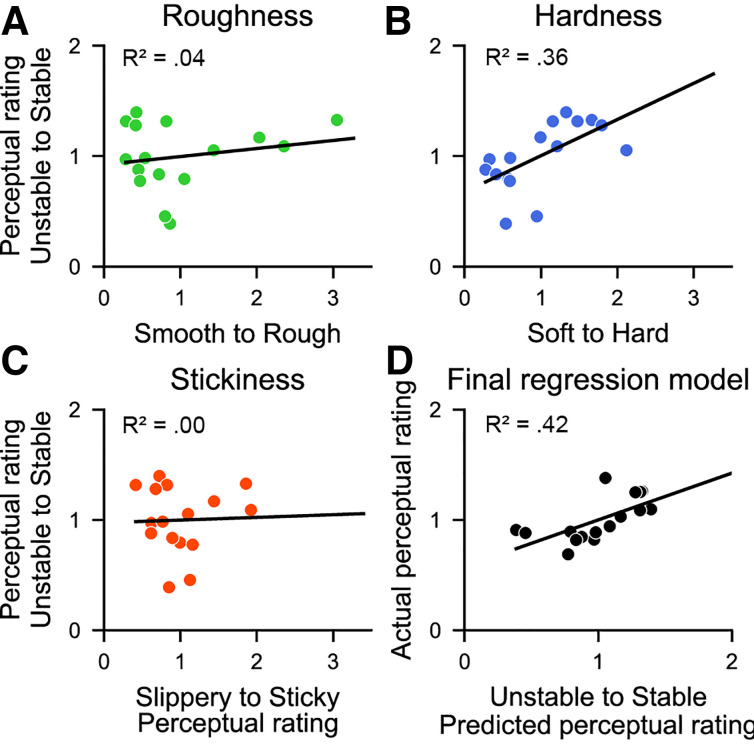
Relationship between textural dimensions and perceived stability. *A*: the relationship between perceived roughness and stability. *B*: the relationship between perceived hardness and stability. *C*: the relationship between perceived stickiness and stability. *D*: predicted stability ratings from the multiple linear regression model compared with recorded ratings of stability. Each point reflects a single texture, showing average scores across all participants. Lines denote lines of best fit.

To investigate whether and how any of the textural dimensions are related to perception of stability, we regressed average perceptual ratings for the different textural dimensions onto the stability ratings for each texture, both independently and jointly. For roughness, stability ratings quickly plateaued with increasing roughness (Fig. 5*A*), leading to low explanatory power (*R*^2^ = 4%). The relationship between hardness and stability appeared linear (Fig. 5*B*), with perceived stability growing with increasing hardness, explaining 36% of the variance in stability ratings. There was no evident relationship between stickiness and perceived stability (Fig. 5*C*; *R*^2^ = 0%). Finally, running a multiple linear regression, using roughness, hardness, and stickiness as input variables and stability as the output variable explained 42% of the variance in stability ratings. However, only hardness contributed significantly (*P* = 0.05).

## DISCUSSION

The current study investigated texture perception at the foot sole, investigating three textural dimensions—under different contact conditions (stepping vs. gentle exploration with the foot sole) and body region (foot vs. hand). On both the group and individual level, perception was highly correlated across all conditions and textural dimensions. In fact, correlations between conditions were stronger than correlations between participants, indicating more consistent ratings within a given participant across interaction mode and body region than between different individuals for the same condition. Nevertheless, a subset of individual textures showed systematic differences across conditions, with the biggest shifts induced by the stepping condition. Texture ratings were also spread more widely in this condition, suggesting that most of the differences in texture perception observed on the foot is likely due to the difference in contact mode rather than an inherent property of this skin site. The results also suggested that hardness is the only perceptual dimension that contributes to participants’ perception of stability, with neither roughness nor stickiness ratings yielding significant correlations.

### Body Region

We found that texture perception was broadly consistent between the hand and the foot, with high correlations between all tested conditions for all three textural dimensions. Indeed, when there were differences, these were rarely grouped by body region, but instead appeared to depend on specific modes of interaction (such as stepping, see further discussion in *Mode of Interaction*). In agreement with these findings, correlations between conditions within the same participants were generally higher than those between participants. At the same time, the between-participant correlations in the present study were comparable with those established previously. For example, Richardson et al. (36) used a comparable set of everyday textures, which they asked participants to explore actively with their hands. The authors found a correlation of *r* = 0.70 in similarity ratings between participants, which the results of the present study are in line with. The observed consistency between body regions is surprising, given the differences in innervation density and skin mechanics between both regions, and might suggest a central mechanism to maintain perceptual constancy across the hairless skin on the body. Future research should further investigate the extent of perceptual constancy across more body regions, extending to hairy skin sites (see Ref. 1).

### Mode of Interaction

We found that changing the mode of interaction, from gentle exploration with the foot sole to stepping onto and off the texture, led to greater changes in texture perception than using a different body region, that is switching from the hand to the foot. This difference was manifested in lower correlations of perceptual ratings in the stepping condition with the other two conditions, but also an expansion in the range of responses for roughness and hardness.

One major difference in the stepping condition was arguably the much higher forces acting on the foot during stepping compared with seated exploration, which might explain some of these differences. Indeed, texture perception on the hand is known to depend on the force applied, with higher forces increasing the perception of roughness (10, 14). However, forces investigated in previous research on the hand are minute compared with those experienced by the foot during everyday behavior, which regularly exceed body mass more than threefold (16). Another difference was that participants were able to use exploratory stroking movements when touching the texture with their hand or the foot when seated. Such active exploration has been shown to influence texture perception on the hand when compared with static presentation, for example for stickiness (37) and roughness (13). Moreover, different types of active exploration, such as stroking or pushing have also been shown to alter perception (38). When walking, deliberate low-force exploratory movements are not possible. However, walking might also not resemble static presentation conditions, because the foot does not touch the ground uniformly and at the same time. Instead, the rolling motion during foot placement (where the heel strikes the ground before the mid-foot) and push off (where the mid-foot leaves the floor before the toes) causes significant shear forces (39, 40), which might provide rich temporal information. Nevertheless, despite the differences seen in the stepping condition, these were relatively small, suggesting that texture perception is broadly similar across all interaction conditions.

### Relating Textural Dimensions to Stability

Humans must be able to maintain balance when walking or standing on range of surfaces to prevent falls and subsequent injury. Textural cues might provide relevant, rapid hints regarding the current surface, and therefore contribute to perceived stability. For example, slippery or very soft surfaces might be perceived as less stable. However, out of the three textural dimensions investigated in the current study, only hardness contributed significantly to perceived stability, explaining ∼40% of total variance in stability ratings, with harder surfaces rated as more stable. Previous research has reported similar decreases in perceived stability in older adults when standing on soft (e.g., foam) rather than hard surfaces (41). Interestingly, when controlling for actual changes in postural sway, older adults who had previously fallen experienced the greatest decreases in perceived stability from hard-to-soft surfaces. The authors suggested that this may have been driven by increased fear of falling experienced by those who had previously fallen, given that experimentally induced fear of falling is known to make people feel less stable (42, 43). Future work should look to explore if these relationships are driven by changes in hardness perception. For instance, do older adults who have fallen rate “hard” textures as softer, and does this underpin the more pronounced changes in perceived stability when standing on soft surfaces? Does fear of falling alter our perception of texture hardness, leading to textures previously perceived as hard to now be experienced as softer? Recently developed virtual reality paradigms (44–46) could help answer these questions.

Neither roughness nor stickiness showed any relationship with perceived stability in the present work. This might suggest that texture only plays a limited part in perceived stability or that these cues are processed in a more complex, perhaps nonlinear way. However, it should be noted that the textures used in the current study did not pose any serious threat to participants’ stability. For example, no extremely rough, unstable (e.g., gravel) or extremely slippery surface (soapy, wet tile) was included in the study. It is therefore possible that texture at the extreme ends of the spectrum does play a greater part in the perceived stability. Nevertheless, even the textures used in the current study yielded a wide range of stability ratings, which could not be fully explained by textural ratings alone. It should be noted that the three dimensions tested here do not fully cover the perceptual texture space and other textural aspects might still contribute to stability.

Recent research has begun to explore the use of textured insoles to improve balance (e.g., see Refs. 24, 25, and 47), following the hypothesis that increased tactile feedback aids balance. Such textured insoles have been shown to reduce postural sway (48), especially when standing on an unstable surface such as foam (22). The majority of these interventions currently focus on using rough textures; as we did not find any relationship between roughness and perceived stability per se, it is likely that these insoles act via a more indirect mechanism, such as generally elevating the level of tactile feedback and subsequent muscle activity (47, 49). Indeed, roughness is highly correlated with neural activity in the hand (28), suggesting that textures perceived as very rough might make good candidates for insoles, taking into account the fact that they will feel even rougher during walking, as shown in this study.

### Limitations

The current study aimed to strike a balance between presenting a diverse set of textures, assessing texture perception along multiple textural dimensions, and allowing for broad comparisons between the foot and the hand, but also different modes of texture interaction of the foot itself. As such, the texture set was necessarily limited. For example, we only used textures that did not pose any serious threat to participants’ stability to ensure the safety of participants and maintain a focus on texture perception. As a consequence, the relationship between texture and stability needs to be interpreted with this limitation in mind and it is possible that more challenging or balance-threatening textures might contribute differently to stability (see discussion aforementioned). The textures were selected as they are commonly encountered in everyday life. However, as a consequence, their properties were not carefully controlled, as is possible with artificial texture sets, and their overall diversity precludes testing the perception of subtly different textures. Although one prior study has investigated the ability to discriminate between similar textures (50), further research is required to get a complete picture regarding the capability of the tactile system on the foot sole. Such future research might also directly compare texture perception on different body parts or via different interactions by presenting participants with pairs of textures during a single trial. However, such experiments are data-intensive; in the present study, our focus was on investigating texture perception on the foot sole as broadly as possible.

Our aim was to focus on conditions that mimic natural interactions with textures as closely as possible (e.g., walking). Although this strategy ensured behavioral relevance, comparisons across conditions need to be interpreted with care. For example, although contact force was a major difference between the stepping and exploration conditions, these also differed in other, potentially relevant, aspects, such as the presence of dynamic motion between the texture and the skin. Future studies might include passive stimulation or better controlled active conditions (such as purely static presentation of the textures or including a high-force condition on the hand) to isolate the contributions of these different effects. A final limitation relates to the lack of objective postural stability measure (e.g., trunk instability when stepping onto or off the textured surfaces). However, as we were interested in how different textures affected perceptual outcomes (rather than posture itself), we do not deem this a major limitation. Furthermore, previous work has shown strong correlations between perceived and objective postural (in)stability outcomes (34, 35)—particularly when eyes are closed (35), as in the present study. Nonetheless, future work should look to explore how texture perceptions interact with objective postural control outcomes.

## DATA AVAILABILITY

All raw data and code used to analyze the data can be found at: https://doi.org/10.17605/OSF.IO/SP8K2.

## GRANTS

L.D.C. was supported by a studentship from the MRC Discovery Medicine North (DiMeN) Doctoral Training Partnership MR/N013840/1. T.J.E. was supported by a Wellcome Trust Sir Henry Wellcome Fellowship 222747/Z/21/Z. H.P.S. was supported by Leverhulme Trust Research Project Grant RPG-2022-031.

## DISCLOSURES

No conflicts of interest, financial or otherwise, are declared by the authors.

## AUTHOR CONTRIBUTIONS

L.D.C., T.J.E., and H.P.S. conceived and designed research; L.D.C., M.R., and C.R.B. performed experiments; L.D.C. analyzed data; L.D.C. and H.P.S. interpreted results of experiments; L.D.C. prepared figures; L.D.C., T.J.E., and H.P.S. drafted manuscript; L.D.C., T.J.E., and H.P.S. edited and revised manuscript.
